# The associations between health-related physical fitness and fasting blood glucose in war veterans: a population-based study

**DOI:** 10.1038/s41598-022-11059-1

**Published:** 2022-04-29

**Authors:** Mario Kasović, Lovro Štefan, Zvonimir Kalčik

**Affiliations:** 1 Department of General and Applied Kinesiology, Faculty of Kinesiology, University of Zagreb, Horvaćanski zavoj 15, 10 000 Zagreb, Croatia; 2 Division of Sport Motorics and Methodology in Kinanthropology, Faculty of Sports Studies, Masaryk University, Brno, Czech Republic; 3 Recrutiment and Examination (RECETOX), Faculty of Science, Masaryk University, Brno, Czech Republic; 4 Home of Croatian Veterans, Zagreb, Croatia

**Keywords:** Endocrinology, Risk factors

## Abstract

The main purpose of the study was to analyze the associations between health-related physical fitness and fasting blood glucose in war veterans. In this cross-sectional study, we recruited 764 men and women aged 45–75 years, who were part of the Homeland War between 1990 and 1995 (33.5% women). Health-related physical fitness included: (1) fat mass and fat-free mass (body composition), (2) push-ups in 30 s (muscular dynamic endurance of upper extremities), (3) sit-ups in 30 s (repetitive upper body strength), (4) chair-stands in 30 s (lower body strength), (5) sit-and-reach test (flexibility) and (6) the 2-min step test (cardiorespiratory function). Laboratory measurement of fasting blood glucose was performed according to standardized procedures in resting seated position after a 12-h overnight fast. Generalized estimating equations with multiple regression models were used to calculate the associations between health-related physical fitness and fasting blood glucose. In men, fasting blood glucose was significantly correlated with fat-free mass (*β* = − 0.25, *p* < 0.001), push-ups in 30 s (*β* = − 0.55, *p* < 0.001), chair-stands in 30 s (*β* = − 0.50, *p* < 0.001), sit-ups in 30 s (*r* = − 0.45, *p* < 0.001), the sit-and reach test (*r* = − 0.46, *p* < 0.001) and the 2-min step test (*r* = − 0.19, *p* < 0.001), while fat mass was positively correlated with fasting blood glucose (*β* = 0.14, *p* = 0.004). In women, fasting blood glucose was significantly correlated with fat mass (*β* = 0.20, *p* = 0.002), fat-free mass (*β* = − 0.15, *p* = 0.014), push-ups in 30 s (*β* = − 0.49, *p* < 0.001), chair-stands in 30 s (*β* = − 0.43, *p* < 0.001), sit-ups in 30 s (*β* = − 0.52, *p* < 0.001), the sit-and reach test (*β* = − 0.40, *p* < 0.001) and the 2-min step test (*β* = − 0.35, *p* < 0.001). This study shows that fasting blood glucose may be predicted by health-related physical fitness test in war veterans.

## Introduction

Blood glucose is one of the essential sources of energy in the body, being critical for optimal health functioning^1^. Studies have shown, that even small increases in blood glucose may lead to negative health-related outcomes, including cardiovascular^2^, metabolic^3^ and eye vision diseases^4^. According to The World Health Organization, 2.2 million deaths were attributable to high blood glucose in 2012 worldwide^5^. Because the levels of blood glucose depend on various factors^6–9^, the measurement is standardized to fasting or resting state^10^.

Although elevated fasting blood glucose has been associated with a wide range of diseases^11,12^, one previous study has shown a nonlinear, J-shaped curve, where both decreased and increased levels of fasting blood glucose may augment the risk of coronary heart disease^7^.

It has been reported, that three-quarter of cardiovascular deaths can be prevented by embracing healthy lifestyle changes^13^, including adequate physical activity. Evidence suggests that regular physical activity may have beneficial effects on blood glucose control^1,13–17^ and glycemic variability^18^. On the other hand, studies using cross section sedentary adults have shown no change^19,20^ or even an increase^17,21^ in fasting blood glucose under the physical activity regime.

Along with physical activity, physical fitness is defined as ‘a measure of the capacity to perform physical activity and/or physical exercise that integrates the majority of the bodily functions (skeletomuscular, cardio-respiratory, hematocirculatory, endocrine-metabolic, and psycho-neurological) involved in bodily movement’^22^. The distinction between physical activity (‘any bodily movement produced by skeletal muscle which requires consumption of energy’)^22^ and physical fitness is crucial, because previous research has shown only a moderate correlation between them^23^. Thus, the associations between physical activity and physical fitness with other health-related outcomes may exhibit different results. The associations between physical fitness and blood glucose have been previously studied^24–28^. The majority of studies have used cardiorespiratory^24–26^ and muscular^27,28^ fitness to explore the associations with blood glucose. In general, lower levels of cardiorespiratory and muscular fitness lead to more impaired blood glucose.

Compared to general population, war veterans are at increased risk for having unstable glucose control and vascular complications^29^. On the other hand, it has been reported that veterans describe challenges in maintaining their physical performance^30,31^. By exploring the associations between various physical fitness components and blood glucose, health-related professionals and trainers may be more aware of which aspect of physical fitness is more protective against high blood glucose and which training mode needs to be incorporated within the rehabilitation center.

Therefore, the purpose of the study was to analyze the associations between health-related physical fitness and fasting blood glucose in war veterans. We hypothesized, that all physical fitness components would be associated with fasting blood glucose, irrespective of sex and age.

## Methods

### Study participants

In this cross-sectional study, we recruited men and women aged 45–75 years, who participated in a homeland war between Croatia and Serbia from 1990 to 1995. More detailed information about the rehabilitation institution and specific programs done with war veterans are described elsewhere^32^. In brief, the Home for Croatian Veterans is a rehabilitation center established by the Ministry of Croatian Veterans specialized in improving quality of life and overall well-being. The users of the Home are accommodated 24 h a day for 21 days. For the purpose of this study, we collected the data from 2017 to 2020 for all of the participants within the facility care. During the period of 4 years, approximately 2500 users used the accommodation service. The inclusion criteria to be part of the study were: 1) being without chronic diseases, 2) having no history of psychiatric symptoms or treatments, 3) being able to perform CRF and MF tests and 4) participating in the Homeland War for at least 100 days. Of 2500, 764 war veterans met the inclusion criteria at baseline and were included in further analyses. By using such sample size, two-tailed test α < 0.05, and the statistical power of 0.95, we would be able to detect a minimum effect size of 0.13 by using G*Power software^33^. All participants eligible for the study had their blood samples collected and were measured in all physical fitness tests. Based on sociodemographic characteristics, the majority of the participants were men (66.5%) with finished secondary education (74.3%), did not participate in regular physical activity of at least moderate intensity (15.4%) and 61.2% were categorized as smokers at the time the study had been conducted. Before the study began, all of the participants gave written informed consent for participation. All of the procedures were anonymous, in accordance with the Declaration of Helsinki^34^ and were followed by the STROBE checklist^35^. also approved by the Ethics Committee of the Home for the Croatian War Veterans(Ethics code number: 2017/04).

### Fasting blood glucose

Blood samples were collected in the morning hours between 8:00 and 10:00 am in resting seated position after a 12-h overnight fast. Each blood sample was drawn from the forearm using a vacutainer blood collection tube with a needle. Laboratory measurement of fasting blood glucose was performed according to standardized procedures^36^.

### Health-related physical fitness

To assess the level of cardiorespiratory fitness, we administrated *the 2-min step test*^37^. The procedure for performing the test has been described previously^37^. In brief, the participant stands up straight next to the wall while a mark is placed on the wall at the level corresponding to midway between the patella (kneecap) and iliac crest (top of the hip bone). When the measurer gives the signal, the participant starts to march in place for two minutes, lifting the knees to the height of the mark on the wall. If the participant needs to rest, it is allowed by holding onto the wall or a stable chair. The test is completed after two minutes of stepping and the result is expressed as the total number of times the right and the left knee reach the tape level in two minutes. Previous evidence confirms an excellent test–retest reliability property (ICC = 0.90, *p* < 0.001) and satisfactory validity, when compared with treadmill performance (*r* = 0.74, *p* < 0.001)^37^. Also, the 2-min step test may successfully detect expected declines across different age groups and significant differences in active *vs.* inactive individuals^37^.

To assess muscular dynamic endurance and ability to stabilize the upper body, *the push-up test in 30 s* was applied^38^. A normal straight leg push-up to straight elbows going laterally was performed, after which the participant pushed down in the initial position. Alternately, if the participants could not complete the push-up test, the test was carried out on the knees. The final score was recorded as the number of push-ups in 30 s.

*The chair stands in 30 s* is the test developed to assess lower body strength^37^. It consists of standing up and sitting down from a chair as many times as possible within 30 s. The participant sat on the standard chair (with an approximate height of 40 cm) with their back in an upright position. Each participant was instructed to look straight forward and to rise with their legs fully extended after the “1, 2, 3, go” command at their own preferred speed with their arms folded across their chest. All trials were performed using the same chair and with similar ambient conditions^39^. The final score involved counting the number of stand-ups in 30 s.

*The sit-up test in 30 s* was performed to assess repetitive upper body strength^40^. The participants were instructed to perform as many correct bent-knee sit-ups as possible in 30 s, while lying on a mat in a supine position with the knees bent at an angle of approximately 90° and keeping the feet together^40^. The arms were put at the chest with the hands-on opposite shoulders. From the initial position, each participant performed a full sit-up to the upright position with their elbows touching their thighs and then returned to the supine position where their shoulders (scapula) touched the mat surface^40^. The performance was scored as having as many correct sit-ups in 30 s.

*The sit-and reach test* was conducted on the floor or a mat, legs straight under the angle of 90º, the person being tested reached forward with the arms (hands overlapping). The distance of reach was measured in centimeters using a measuring non-elastic tape attached on the floor^41^. To assess body composition, we used bioelectrical impedance analysis (Omron BF500 Body Composition Monitor, Omron Medizintechnik). The device uses eight electrodes and requires the participant to stand on metal footpads in bare feet and grasp a pair of electrodes fixed on a handle with arms extended in front of the chest^42^. The manufacturer’s pre-programmed equations were used to predict fat mass and fat-free mass. All participants were instructed not to consume food or water before the testing. The same equipment was used for each participant. Standing height and weight were measured following the instruction from previous studies^43^ by using Seca portable 202 scales (Seca, Hamburg, Germany) and a digital scale (Seca, model 769).

Prior the study, technics of sport were instructed, how to conduct the tests. All tests were assessed in the first week of their rehabilitation period. To avoid fatigue, the protocol was split into three non-consecutive days during the morning hours between 8:00 am and 11:00 am. On the first day of measurement, blood samples between 8:00 am and 10:00 am were taken from each participant. On the second day, the tests were performed in following order: 1) fat mass and fat-free mass, 2) the push-up test in 30 s, 3) the chair stands in 30 s, 4) the sit-up test in 30 s and 5) sit-and-reach test. Before every test, the participants had ≈15 min of resting period. On the second day, the 2-min step test was applied.

### Data analysis

Basic descriptive statistics are presented as mean ± SD. Sex differences were examined using the analysis of covariance (ANCOVA), adjusted for age for normally and Man-Whitney test for not normally distributed variables. Cohen d effect sizes (ES) were calculated to determine the magnitude of the sex differences. ES was classified as trivial (< 0.2), small (0.2–0.6), moderate (0.6–1.2), large (1.2–2.0), very large (> 2.0) and extremely large (> 4.0)^44^. Pearson coefficients of correlation (*r*) and generalized estimating equations with multiple regression models were used to calculate the associations between physical fitness and fasting blood glucose. According to Cohen^45^, correlations were considered as weak (0.10), moderate (0.30) and strong (0.50). We tested the data for multicollinearity using the variance inflation factors, normality of residuals using the normal probability plot and histogram of residuals and heteroscedasticity using the standardized residuals versus predicted plot. The variance inflation factors in our model ranged from 1.53 to 2.49 indicating no multicollinearity and the other assumptions were also met. In an unadjusted model (Model 1), we calculated separate associations between various physical fitness components and fasting blood glucose. In Model 2, all physical fitness components were put simultaneously, adjusted for sex and age. The results are presented as *β* coefficients. All analyses were performed in Statistical Packages for Social Sciences version 23. (SPSS Inc., Chicago, IL, USA) with statistical significance of *p* ≤ 0.05.

### Ethics approval

Ethical Committee of The Home for Croatian Veterans (Ethics code number: 2017/04).

### Consent to participate

The informed consent voluntarily was signed by the participants.

## Results

Basic descriptive statistics of the study participants are presented in Table 1. Men were significantly taller, heavier, and had lower percentage of fat mass and higher percentage of fat-free mass, compared to women. Men also performed better in muscular fitness tests, while women exhibited higher results in flexibility. No significant differences in cardiorespiratory function nor fasting blood glucose were observed.

**Table 1:** Basic descriptive statistics of the study participants (*N* = 764).

	Total (*N* = 764)	Men (*N* = 508)	Women (*N* = 256)	Cohen’s *D*	*p* value†
	Mean ± SD	Mean ± SD	Mean ± SD		
Age (years)	59.9 ± 7.6	60.0 ± 7.8	59.9 ± 7.1	0.01	0.878
Stature (cm)	172.5 ± 9.1	177.1 ± 6.7	163.5 ± 5.9	2.03	< 0.001
Body mass (kg)	90.3 ± 18.5	96.2 ± 22.2	78.3 ± 14.7	0.81	< 0.001
Body-mass index (kg/m^2^)	29.7 ± 5.5	29.8 ± 5.9	29.4 ± 5.0	0.07	0.303
Fat mass (%)	27.7 ± 12.9	23.9 ± 11.0	35.0 ± 13.3	1.01	< 0.001
Fat-free mass (%)	27.0 ± 11.3	28.1 ± 11.2	25.0 ± 11.3	0.28	< 0.001
Fasting glucose (mmol/L)	5.8 ± 1.3	5.8 ± 1.3	5.9 ± 1.3	0.08	0.610
Push-ups in 30 s (reps)	9.7 ± 4.3	10.2 ± 4.9	8.7 ± 3.9	0.31	0.008
Chair-stands in 30 s (reps)	11.4 ± 4.8	12.0 ± 5.1	10.1 ± 3.9	0.37	< 0.001
Sit-ups in 30 s (reps)	9.4 ± 3.2	9.9 ± 3.3	8.4 ± 3.0	0.45	0.006
2-min step test (reps)	111.6 ± 19.7	111.4 ± 20.1	112.0 ± 19.0	0.03	0.722
Sit-and-reach test (cm)	42.6 ± 16.4	39.5 ± 16.2	48.7 ± 15.1	0.57	< 0.001

^†^Denotes using the analysis of covariance (ANCOVA) for normally distributed or Man–Whitney *Z*-test for not normally distributed variables; *p* < 0.05.

In men, partial Pearson’s coefficient of correlation adjusted for age and body-mass index showed that higher levels of fasting blood glucose were significantly correlated with lower values in fat-free mass (*r* = − 0.25, *p* < 0.001), push-ups in 30 s (*r* = − 0.56, *p* < 0.001), chair-stands in 30 s (*r* = − 0.54, *p* < 0.001), sit-ups in 30 s (*r* = − 0.50, *p* < 0.001), the sit-and reach test (*r* = − 0.54, *p* < 0.001) and the 2-min step test (*r* = − 0.25, *p* < 0.001), while fat mass was positively correlated with fasting blood glucose (*r* = 0.15, *p* = 0.011). In women, partial Pearson’s coefficient of correlation adjusted for age and body-mass index showed that higher levels of fasting blood glucose were significantly correlated with lower values in push-ups in 30 s (*r* = − 0.50, *p* < 0.001), chair-stands in 30 s (*r* = − 0.47, *p* < 0.001), sit-ups in 30 s (*r* = − 0.58, *p* < 0.001), the sit-and reach test (*r* = − 0.49, *p* < 0.001) and the 2-min step test (*r* = − 0.40, *p* < 0.001), while no significant correlations with fat-free mass (*r* = − 0.10, *p* = 0.254) and fat mass (*r* = 0.01, *p* = 0.987) were observed.

Table 2 shows the associations between fasting blood glucose and physical fitness in men and women. In the unadjusted model (Model 1), all physical fitness components were negatively associated with fasting blood glucose, except for fat mass, where a positive association was found. When the model was adjusted for age and body-mass index (Model 2), all physical fitness components remained significantly associated with fasting blood glucose in both genders and with similar direction of the association. Of note, a multiple regression model showed, that all seven physical fitness tests were highly and similarly associated with fasting blood glucose in men (*R* = 0.77, *R*^*2*^ = 0.59, *standard error of the estimate* = 0.89 mmol/L, *p* < 0.001) and women (*R* = 0.77, *R*^*2*^ = 0.59, *standard error of the estimate* = 0.88 mmol/L, *p* < 0.001).

**Table 2:** The associations between health-related physical fitness and fasting blood glucose in the study participants, stratified by gender (*N* = 764).

Study variables	Model 1				Model 2			
	*β*	*95% CI*	*t* value	*p* value	*β*	95% CI	*t* value	*p* value
**Men (*N* = 508)**								
Fat mass (%)	0.09	− 0.18 to 0.01	1.91	0.057	0.14	0.05 to 0.24	2.90	0.004
Fat-free mass (%)	− 0.25	− 0.34 to 0.16	− 5.67	< 0.001	− 0.25	− 0.33 to 0.16	− 5.77	< 0.001
Push-ups in 30 s (reps)	− 0.59	− 0.73 to 0.55	− 13.89	< 0.001	− 0.55	− 0.70 to 0.51	− 12.23	< 0.001
Chair-stands in 30 s (reps)	− 0.54	− 0.64 to 0.47	− 12.97	< 0.001	− 0.50	− 0.62 to 0.44	− 11.81	< 0.001
Sit-ups in 30 s (reps)	− 0.49	− 0.64 to 0.44	− 10.46	< 0.001	− 0.45	− 0.60 to 0.39	− 9.54	< 0.001
2-min step test (reps)	− 0.24	− 0.32 to 0.15	− 5.27	< 0.001	− 0.19	− 0.27 to 0.10	− 4.09	< 0.001
Sit-and-reach test (cm)	− 0.49	− 0.60 to 0.42	− 11.29	< 0.001	− 0.46	− 0.58 to 0.40	− 10.64	< 0.001
**Women (*N* = 256)**								
Fat mass (%)	0.04	− 0.08 to 0.18	0.67	0.503	0.20	0.07 to 0.32	3.13	0.002
Fat-free mass (%)	− 0.19	− 0.31 to 0.07	− 3.01	0.003	− 0.15	− 0.27 to 0.03	− 2.47	0.014
Push-ups in 30 s (reps)	− 0.54	− 0.70 to 0.40	− 8.51	< 0.001	− 0.49	− 0.64 to 0.38	− 7.80	< 0.001
Chair-stands in 30 s (reps)	− 0.46	− 0.60 to 0.35	− 7.43	< 0.001	− 0.43	− 0.57 to 0.32	− 7–17	< 0.001
Sit-ups in 30 s (reps)	− 0.57	− 0.74 to 0.48	− 9.17	< 0.001	− 0.52	− 0.69 to 0.43	− 8.75	< 0.001
2-min step test (reps)	− 0.37	− 0.51 to 0.25	− 5.67	< 0.001	− 0.35	− 0.49 to 0.22	− 5.23	< 0.001
Sit-and-reach test (cm)	− 0.45	− 0.59 to 0.34	− 7.23	< 0.001	− 0.40	− 0.53 to 0.28	− 6.45	< 0.001

**Model 1:** examines the associations between each health-related physical fitness test entered separately into the model and fasting blood glucose.

**Model 2:** examines the associations between each health-related physical fitness test entered separately into the model and fasting blood glucose adjusted for age and body-mass index.

## Discussion

The main purpose of the study was to analyze the associations between health-related physical fitness and fasting blood glucose in war veterans. We found significant inversed associations between fasting blood glucose and all physical fitness components, except for positive association with fat mass. If all physical fitness components were used simultaneously, fasting blood glucose accounted for 60% of the variance.

In the regression model, we found that fat mass was positively associated with fasting blood glucose, yet negative association between fat-free mass and fasting blood glucose was observed. Such results have been confirmed previously^46–49^. Specifically, a study by Kim & Park^46^ showed that compared to high muscle and low-fat group, those participants categorized as having low muscle and high fat and high muscle and high fat were 1.90 and 2.30 more likely to develop metabolic syndrome. However, a follow-up study by Kim et al*.*^49^ highlighted that only changes in total body fat mass were associated with development of Type 2 diabetes, while no significant associations between the changes in fat-free mass and Type 2 diabetes were observed. Finally, a large cohort study examining the associations between fat-free mass and insulin resistance concluded that fat-free mass was an independent predictor of insulin resistance^49^.

Our findings of the associations between fasting blood glucose and cardiorespiratory fitness are in line with previous studies^24–28^. For example, a study by Loprinzi and Pariser^24^ showed that cardiorespiratory fitness assessed through a submaximal treadmill-based test was significantly associated with impaired blood glucose control in obese and inactive adults. Another cross-sectional study presented similar results, where a low maximal oxygen uptake was moderately associated with high fasting blood glucose (*r* = − 0.34)^26^. Similar associations have been obtained in previous longitudinal studies^25^. During an average follow-up of 6 years, men in the low-fitness group (the least fit 20% of the cohort) at baseline were almost two times more likely to have impaired blood glucose, compared to with those in the high-fitness group (the most fit 40% of the cohort)^25^. On the other hand, studies have also shown that muscular fitness may have beneficial effects on glycemic control^28^. In a recent meta-analysis, groups who performed a muscular training lowered glycosylated hemoglobin (mean ES = − 0.37, 95% CI − 0.55 to − 0.20, *p* < 0.01), compared to those who did not receive a treatment. Moreover, the same study showed that high-intensity muscular training groups had a slight tendency to improve glycemic control, irrespective of duration, frequency, and weekly volume^28^.

The mechanism underlying the aforementioned associations in our context may be explained by acute and regular exercise training^10^. For example, exercise may acutely increase muscle glucose transport^50^, which can lead to reverse impaired insulin sensitivity and a reduced endogenous glucose production^51^. Moreover, higher physical performance is associated with induced adaptations of pancreatic β-cells, leading to a reduction in glucose-induced insulin secretion^20^.

This study has a few strengths. First, the findings of the study were based on a large representative sample of war veterans aged 45–75 years. Second, we covered all aspects of physical fitness, including cardiorespiratory, muscular and flexibility fitness and body composition.

However, this study is not without limitations. By using a cross-sectional design, we cannot establish the causality of the association, where higher fasting blood glucose led to lower physical fitness values. Next, cardiorespiratory fitness was assessed by the 2-min step test, which was only moderately correlated with the treadmill protocol and might underestimate the level of ‘real’ cardiorespiratory fitness. Finally, we did not adjust for genetical and environmental factors, which might be associated to both physical fitness and fasting blood glucose. For example, no information on women with menopause was collected. Previous evidence has suggested that postmenopausal women may have lower physical fitness performance, when compared to the premenopausal women^52^. Therefore, future research among war veterans should focus in exploring longitudinal associations between physical fitness and fasting blood glucose with additional adjustments, to establish bidirectional associations between physical fitness and fasting blood glucose.

In conclusion, this study confirms the findings of previous studies, where fat-free mass, cardiorespiratory and muscular fitness are negatively associated, and fat mass is positively associated with fasting blood glucose. Of all physical fitness components in the regression model, cardiorespiratory fitness exhibits the most protective effects against having high fasting blood glucose, followed by muscular dynamic endurance of upper extremities and fat-free mass. Therefore, randomized controlled trials consisted of both aerobic and resistance training protocols should be implemented within the rehabilitation centers.

## Data Availability

The datasets used and/or analyzed during the current study are available from the corresponding author on reasonable request.
